# Draft Genome Sequence of *Limnospira* sp. Strain BM01, Isolated from a Hypersaline Lake of the Momela Ecosystem in Tanzania

**DOI:** 10.1128/MRA.00132-21

**Published:** 2021-04-22

**Authors:** Reuben Maghembe, Angelina Michael, Ajith Harish, Stephen S. Nyandoro, Sylvester L. Lyantagaye, Rajni Hati-Kaul

**Affiliations:** a Department of Molecular Biology and Biotechnology, College of Natural and Applied Sciences, University of Dar es Salaam, Dar es Salaam, Tanzania; b Division of Biotechnology, Center for Chemistry and Chemical Engineering, Lund University, Lund, Sweden; c Department of Biology, College of Natural and Mathematical Sciences, University of Dodoma, Dodoma, Tanzania; d Department of Animal Breeding and Genetics, Swedish University of Agricultural Sciences, Uppsala, Sweden; e Chemistry Department, College of Natural and Applied Sciences, University of Dar es Salaam, Dar es Salaam, Tanzania; f Department of Biochemistry, Mbeya College of Health and Allied Sciences, University of Dar es Salaam, Mbeya Tanzania; Georgia Institute of Technology

## Abstract

The genus *Limnospira* includes cyanobacterial species used for industrial production of dietary supplements and nutraceutical agents. The metagenome-assembled genome of *Limnospira* sp. strain BM01 from Big Momela Lake, Tanzania, was 6,228,312 bp long with a GC content of 44.8% and carried 4,921 proteins and 52 RNA genes, including 6 rRNA genes.

## Abstract (precis)

The genus *Limnospira* includes cyanobacterial species used for industrial production of dietary supplements and nutraceutical agents. The metagenome-assembled genome of *Limnospira* sp. strain BM01 from Big Momela Lake (BM), Tanzania, was 6,228,312 bp long with a GC content of 44.8% and carried 4,921 proteins and 52 RNA genes, including 6 rRNA genes.

## ANNOUNCEMENT

*Limnospira* is a newly described genus of cyanobacteria. This genus currently includes three commercially important species, which belonged previously to the genus *Arthrospira* (1). *Arthrospira* and *Limnospira* species are well known for their applications ranging from food supplements to industrial polymers (2–4). The East African Rift Valley Region contains a number of saline lakes that are among the most productive ecosystems in the world (5, 6), albeit with very few studies being done there (6, 7). As a part of our investigations on identifying the genomes of potentially industrial halophiles constituting part of the Big Momela microbiome, we report here the first draft genome sequence of a *Limnospira* species from that ecosystem.

The strain BM01 was obtained from Big Momela Lake in Arusha, Tanzania (3.2242°S, 36.9098°E), an alkaline ecosystem with a pH range of 8 to 10. Water samples were taken in 2019 at a depth of 1 m from the lake by dipping a 10-liter bucket. The samples were then filtered through a plankton net of 20-μm mesh size, enriched with a standard Zarrouk medium (7), and incubated at 26°C for 5 days to exclude the nonhalophiles. This primary culture was further enriched by incubating it at 28°C for 6 additional days.

Metagenomic DNA was extracted using a ZymoBIOMICS DNA miniprep kit (ZR D4300) following the manufacturer’s protocol. Illumina paired-end libraries were constructed from 3.136 μg of DNA using a TruSeq DNA PCR-free kit and TruSeq Nano DNA kit. DNA sequence reads of 150 bp average length generated from the Illumina NovaSeq pipeline were quality controlled using FastQC v0.11.5 and filtered with Trimmomatic v0.36 before assembly.

Draft metagenomic contigs were generated by *de novo* assembly using SPAdes v3.14.1 and then annotated with MG-RAST v4.0.3 (8) to obtain genus/species-level assignments. Since contigs assigned to *Arthrospira*/*Limnospira* were the most abundant (76.38%), the metagenome contigs were mapped directly against eight *Arthrospira*/*Limnospira* genomes available in NCBI GenBank with CONTIGuator v2.7.5, using default parameters (9). Of all the retrieved reference genomes, Limnospira indica PCC 8005 (GenBank accession number FO818640.1) emerged as the most appropriate reference genome with the best mapping. This reference-guided assembly had a GC content of 44.8% and *N*_50_ and *L*_50_ values of 51,800 bp and 38, respectively.

The draft genome sequence was annotated using the Prokaryotic Genome Annotation Pipeline (PGAP) (https://www.ncbi.nlm.nih.gov/genome/annotation_prok/). The draft genome sequence contained a total of 5,566 coding sequences (CDS); 4,921 were identified as protein-coding sequences, 52 were RNA coding genes (i.e., 42 tRNA genes, 6 rRNA genes, and 4 noncoding RNAs [nc-RNAs]), and 3 were CRISPR arrays.

### Data availability.

This draft genome sequence is available under the accession number CP060212.1, BioProject accession number PRJNA648758, locus tag prefix H2674, and BioSample number SAMN15646860. The raw reads for the metagenomic assembly (MAG) are available under the SRA number SRX9970723.
